# Impact of DNA repair gene polymorphisms on the risk of biochemical recurrence after radiotherapy and overall survival in prostate cancer

**DOI:** 10.18632/oncotarget.15282

**Published:** 2017-02-11

**Authors:** Chiara Zanusso, Roberto Bortolus, Eva Dreussi, Jerry Polesel, Marcella Montico, Erika Cecchin, Sara Gagno, Flavio Rizzolio, Mauro Arcicasa, Giacomo Novara, Giuseppe Toffoli

**Affiliations:** ^1^ Experimental and Clinical Pharmacology Unit, National Cancer Institute, Aviano, PN, Italy; ^2^ Department of Radiation Oncology, National Cancer Institute, Aviano, PN, Italy; ^3^ Epidemiology and Biostatistics Unit, National Cancer Institute, Aviano, PN, Italy; ^4^ Department of Surgery, Oncology, and Gastroenterology, University of Padua, Padua, Italy

**Keywords:** prostate cancer, polymorphisms, biochemical recurrence, overall survival, DNA repair

## Abstract

The identification of biomarkers of biochemical recurrence (BCR) in prostate cancer (PCa) patients undergoing radiotherapy (RT) represents an unanswered clinical issue. The primary aim of this study was the definition of new genetic prognostic biomarkers in DNA repair genes (DRGs), considering both BCR and overall survival (OS) as clinical end-points. The secondary aim was to explore the potential clinical impact of these genetic variants with the decision curve analysis (DCA) and the sensitivity analysis.

We analyzed 22 germline polymorphisms in 14 DRGs on 542 Caucasian PCa patients treated with RT as primary therapy. Significant associations were further tested with a bootstrapping technique. According to our analyses, *ERCC2*-rs1799793 and *EXO1*-rs4149963 were significantly associated with BCR (*p* = 0.01 and *p* = 0.01, respectively). Moreover, *MSH6*-rs3136228 was associated with a worse OS (*p* = 0.04). Nonetheless, the DCA and the sensitivity analyses gave no ultimate response about the clinical impact of such variants.

This study highlights the potential prognostic role of polymorphisms in DRGs for PCa, paving the way to the introduction of not invasive tools for the personalization of patients management. Nonetheless, other prospective studies are necessary to ultimately clarify the clinical impact of pharmacogenetics in PCa.

## INTRODUCTION

The clinical course of prostate cancer (PCa) patients is difficult to predict since men with similar tumour features can experience strikingly different outcomes. Despite the improvement obtained with the introduction of primary radical radiotherapy (RT), either alone or in combination with hormone therapy (HT) [1], a not negligible group of PCa patients (15% to 46%) treated with RT experience biochemical recurrence (BCR). There is a strict association between BCR and other prognostic parameters like overall survival (OS) and risk to develop metastasis [2, 3]. The prompt identification of this subgroup of patients can play a pivotal clinical role because it can be translated in a more frequent patients’ follow-up and a more appropriate maintenance therapy.

Many efforts have been done in order to identify reliable prognostic biomarkers. Some clinical-pathological indicators, like PSA, Gleason score, and TNM stage, are currently used to predict outcome following RT for localized PCa. Nonetheless, the need of more specific and accurate prognostic biomarkers has not been yet overcome [4]. In this scenario, pharmacogenetics could be the key to find an answer to this compelling necessity. Indeed, the effect of RT, in terms of efficacy and toxicity, can be widely influenced by polymorphisms localized in genes coding for DNA repair enzymes [5, 6]. The complex system of DNA repair could be of great interest considering its pivotal role in maintaining genomic integrity. Several analyses have been conducted until now to determine the potential clinical role of polymorphisms in DNA repair genes (DRGs) in patients undergoing RT [7–13]. However, the study of the prognostic role of these genetic variants in terms of both BCR and OS has given till now not conclusive results. Moreover, the potential clinical impact of introducing genetic analysis is not still clear.

Thus, the main aim of this study was to elucidate the potential association of genetic polymorphisms localized in DRGs with BCR and OS studying a large group of PCa patients. To this purpose, we have analyzed the potential prognostic role of twenty-two polymorphisms in fourteen DRGs in a group of 542 Caucasian PCa patients who underwent RT as primary therapy.

The secondary aim of this study was to explore the potential clinical utility of these genetic variants with the application of the decision curve analyses (DCA) and the sensitivity analysis.

## RESULTS

### Patients’ characteristics and clinical outcome

Patients’ clinical and pathological data (age at diagnosis, serum PSA levels at diagnosis and three months after the end of RT, Gleason score, clinical tumor stage defined according to the TNM scale -cT-, first line treatment parameters -RT dosage, HT administration-, date of diagnosis, date of BCR, date of last follow-up or death) were collected from the medical records. More in detail, according to European guidelines, BCR has been defined with an increase of serum PSA levels more than 2 ng/mL above the lowest level reached after the end of RT.

The clinical pathological characteristics of the enrolled patients are shown in Table 1. At the moment of diagnosis, 366 patients (67.5%) presented a locally advanced malignancy (T = 1–2). In 302 patients (55.7%) the Gleason score fluctuated between 2 and 6.

**Table 1:** Clinical and pathological characteristics of enrolled prostate cancer patients

Characteristics	Study population *n* (%)
*N° of subjects*	542
*Age at diagnosis, yr*	
Median	70
IQR	66–73
*Death*	117 (21.6)
*Time to death follow-up time, mo^#^*	
Median (IQR)	67 (45–94)
*PSA at diagnosis^#^, ng/ml*	
Median (IQR)	8.9 (6.0–16.0)
< 7	180 (33.2)
7–13	193 (35.6)
≥ 13	169 (31.2)
*Gleason score^#^*	
2–6	302 (55.7)
7	129 (23.8)
8–10	111 (20.5)
*T stage (TNM scale)*	
T1-T2	367 (67.7)
T3	173 (31.9)
T4	2 (0.4)
*Treatment^#^*	
RT	76 (14.0)
RT+HT	466 (86.0)
*RT dose,Gy^#^*	
≤ 70	47 (8.7)
> 70	495 (91.3)
BCR*	113 (21.3)
*BCR after RT follow-up time, mo*	
Median (IQR)	45 (22–70)

Abbreviations: RT = radiotherapy; HT = hormone therapy; BCR = biochemical recurrence; PSA = prostate-specific antigen; IQR = Inter-quartile range.

*data available for 530 patients.

*^#^*data available for the entire study population (542 patients).

### Genotyping analyses

The average genotype call rate was 99.51% (range: 98.89–100.00%). Two SNPs, *ERCC2*-rs1799793 and *XRCC1*-rs25489, were not in Hardy-Weinberg equilibrium, (*p* = 0.028 and *p* = 0.005, respectively). These SNPs have been associated with prostate cancer risk [14, 15]. Consequently, the deviations could be associated with the cancer onset. We had thus not excluded them from the analysis.

### Clinical-pathological features, germ-line polymorphisms and BCR

The BCR was calculated from the end of RT to relapse. To evaluate BCR risk, twelve patients were excluded due to the lack of the information related to PSA levels after the end of RT. Thus, for this analysis, 530 patients were selected from the complete population of study (542 patients). The median follow-up of this group was 45 months (inter-quartile range (IQR): 22–70 months). One hundred thirteen patients (21.3%) relapsed with a median relapse time of 29 months (IQR: 15–49 months). The overall 5- and 10-yr BCR survival estimates were 76.5% and 54.2%, respectively.

Table 2 summarizes univariate and multivariate analysis for prediction of BCR following RT. In the univariate and in the multivariate Cox proportional hazard model, after adjustment for age, Gleason score, and PSA at diagnosis, *ERCC2-*rs1799793 and *EXO1-*rs4149963 were independently associated with BCR after RT.

**Table 2:** Univariate and multivariate Cox proportional hazards analysis of factors associated with biochemical recurrence after radiotherapy

	BCR (*n* = 530)					
	Univariate		Multivariate		Bootstrap analysis	
	HR (95% CI)	*p*-value	HR (95% CI)	*p*-value	HR (95% CI)	*p*-value
*Age at diagnosis, yr*						
Continuous	0.95 (0.93–0.98)	**0.0027**	0.96 (0.93–0.99)	**0.0083**		
*Gleason score*						
2–6 (reference)						
7	1.70 (1.10–2.64)	**0.0195**	1.53 (0.95–2.45)	**0.0798**		
8–10	1.96 (1.25–3.08)	**0.0035**	1.46 (0.88–2.40)	**0.1414**		
*PSA at diagnosis, ng/ml*						
Continuous	1.011 (1.008–1.014)	**< 0.0001**	1.007 (1.003–1.011)	**0.0006**		
***ERCC2* –rs1799793** Dominant model	0.59 (0.40–0.78)	**0.0060**	0.57 (0.39–0.85)	**0.0051**	0.58 (0.39–0.89)	0.0121
***EXO1*–rs4149963** Dominant model	1.69 (1.09–2.62)	**0.0198**	1.91 (1.21–2.99)	**0.0050**	1.85 (1.16–2.98)	0.0099

Abbreviations: BCR = biochemical recurrence; PSA = prostate–specific antigen; HR = hazard ratio; CI = confidence interval; ERCC2 = Excision Repair Cross–Complementing 2; EXO1 = exonuclease 1

* bootstrap resampling method by drawing 1000 samples from the original dataset

Significant associations (*p* < 0.05) are reported in bolt.

**Figure 1:**
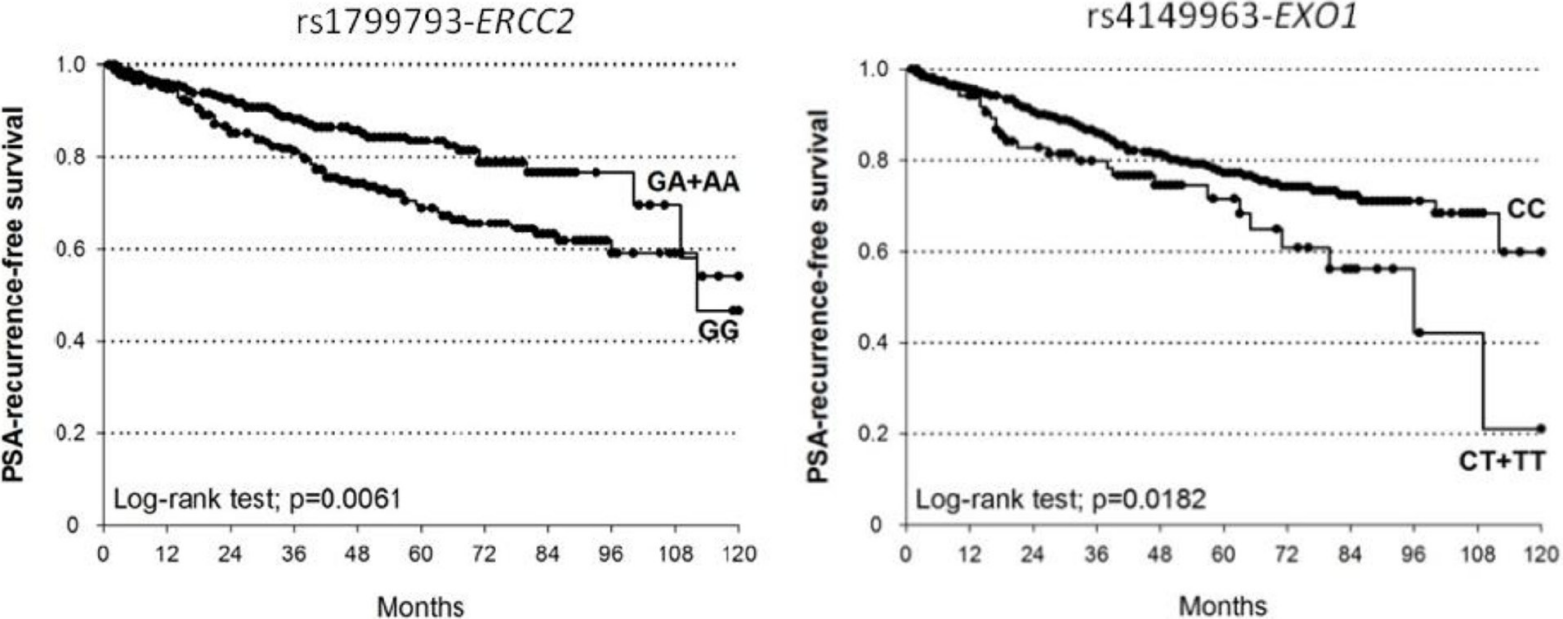
Kaplan-Meier estimates of BCR after RT at 10 years stratified according to genotypes of *ERCC2*-rs1799793 (median survival: GA+AA=not reached; GG=112 months) and *EXO1*-rs4149963 (median survival: CT+TT = 96 months; CC = not reached)

The probability not to experience BCR at 5- and 10-yr for patients bearing at least one A allele of *ERCC2-*rs1799793 patients was 83.6% and 54.2% respectively, whereas in patients carrying *ERCC2-*rs1799793 GG genotype was 69.1% at 5 years and 46.5% at 10 years (Log-rank test *p* = 0.0061; Figure 1). This polymorphism showed a protective role on BCR increasing the probability of PSA-free survival (dominant model: HR = 0.57, 95%CI = 0.39–0.85, *p* = 0.0051).

The median BCR-free survival at 5- and 10-yr for the *EXO1-*rs4149963 CT+TT patients were 71.6% and 21.1% respectively, while for CC genotype were 77.3% and 59.9% (Log-rank test *p* = 0.0182). According to the dominant genetic model, patients carrying at least one variant T allele of *EXO1-*rs4149963 showed an increased BCR risk after RT (HR = 1.91, 95%CI = 1.21–2.99, *p* = 0.0050).

We performed a bootstrap resampling of the significant SNPs by drawing 1000 samples from the original dataset. After this analysis, *ERCC2-*rs1799793 and *EXO1-*rs4149963 still remained significant (dominant model: HR = 0.58, 95%CI = 0.39–0.89, *p* = 0.0121; and dominant model: HR = 1.85, 95%CI = 1.16–2.98, *p* = 0.0099, respectively), reinforcing our data (Table 2).

Finally, a sensitivity analysis was performed in order to evaluate the strength of the obtained markers. Specifically, we have evaluated the capability of *ERCC2-*rs1799793 and *EXO1-*rs4149963 to predict BCR in three different subgroups of patients defined according to the treatment they underwent. The SNP *ERCC2-*rs1799793 maintained its significance in all subgroups, whereas *EXO1-*rs4149963 was significant only for the subgroup characterized by a RT dosage > 70 Gy, suggesting a potential role of HT for this polymorphism (Table 3).

**Table 3:** Sensitivity analyses performed in four groups of patients for BCR and OS

	BCR							
	Group 1: all patients		Group 2: RT+HT		Group 3: RT (> 70 Gy)		Group 4: RT(> 70 Gy)+HT	
	*n* = 530		*n* = 454		*n* = 483		*n* = 425	
	HR (95% CI)	*p*-value	HR (95% CI)	*p*-value	HR (95% CI)	*p*-value	HR (95% CI)	*p*-value
***ERCC2*-rs1799793** Dominant model	0.57 (0.93–0.85)	**0.0051**	0.56 (0.36–0.87)	**0.0105**	0.56 (0.36–0.86)	**0.0078**	0.51 (0.32–0.82)	**0.0047**
***EXO1*-rs4149963** Dominant model	1.91 (1.21–2.99)	**0.0050**	1.70 (1.00–2.92)	0.0517	1.78 (1.09–2.91)	**0.0209**	1.64 (0.93–2.91)	0.0907

	OS							
	Group 1: all patients		Group 2: RT+HT		Group 3: RT (> 70 Gy)		Group 4: RT (> 70 Gy)+HT	
	*n* = 542		*n* = 466		*n* = 495		*n* = 437	
	HR (95% CI)	*p*–value	HR (95% CI)	*p*–value	HR (95% CI)	*p*–value	HR (95% CI)	*p*–value
***MSH6*-rs3136228** Dominant model	0.63 (0.43–0.92)	**0.0163**	0.60 (0.39–0.92)	**0.0191**	0.69 (0.46–1.04)	0.0744	0.72 (0.45–1.15)	0.1659

Abbreviations: RT = radiotherapy; HT = hormone therapy; BCR = biochemical recurrence; OS = overall survival; HR = hazard ratio; CI = confidence interval; ERCC2 = Excision Repair Cross-Complementing 2; EXO1 = exonuclease 1; MSH6 = mutS homolog 6.

1) all eligible patients; 2) patients treated with RT and HT (RT+HT); 3) patients treated with RT dose > 70 Gy (RT(> 70 Gy)); 4) patients treated with RT (> 70 Gy) and HT (RT(> 70 Gy)+HT). Significant associations (*p* < 0.05) are reported in bolt.

**Figure 2:**
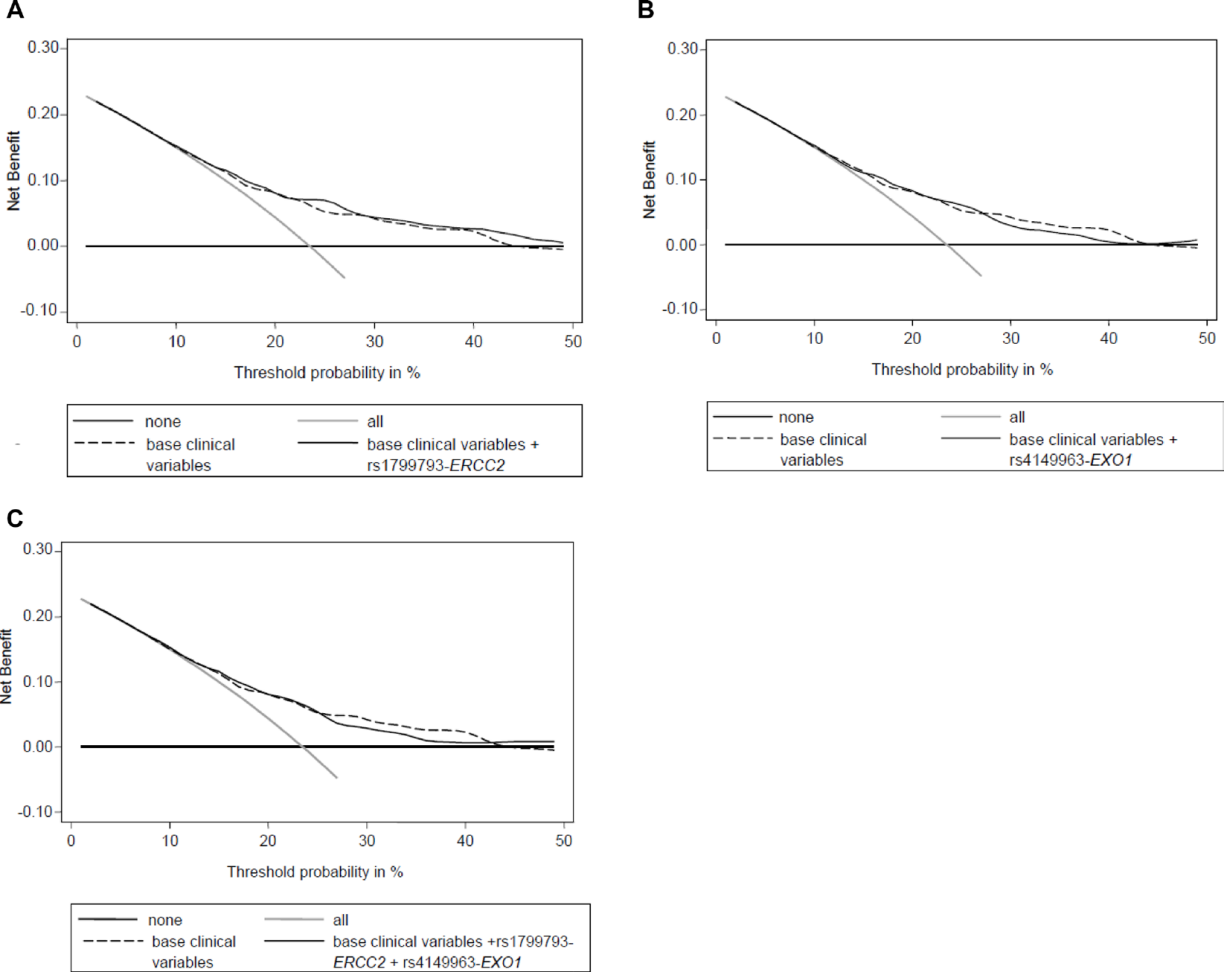
Decision curve analysis for BCR at 5 years including (**A**) base clinical variables (Gleason score, serum PSA level at diagnosis, age at diagnosis, TNM stage, and RT dose (Gy)) and *ERCC2*-rs1799793; (**B**) base clinical variables and *EXO1***-**rs4149963; (**C**) base clinical variables and *ERCC2*-rs1799793 and *EXO1*-rs4149963. “none”: no prognostic analyses are performed; “all”: hypothetical condition when you correctly define all patients that experience relapse; “base”: only clinical variables are analyzed; “base+*ERCC2*”: clinical variables and *ERCC2*-rs1799793 are analyzed; “base+*EXO1*”: clinical variables and *EXO1*-rs4149963 are analyzed; “base+*ERCC2*+*EXO1*”: clinical variables, *ERCC2*-rs1799793 and *EXO1*-rs4149963 are analyzed.

In addition, we performed a (DCA) to evaluate the reliability of predictions performed considering known prognostic clinical variables (Gleason score, serum PSA level at diagnosis, age at diagnosis, TNM stage, and RT dose -Gy-)) and of predictions that take into account both clinical variables and polymorphisms (*ERCC2-*rs1799793 and *EXO1-*rs4149963), either alone or in combination. This analysis was performed evaluating 5-yr BCR. The DCA showed a slight benefit in incorporating both *EXO1-*rs4149963 and *ERCC2*-rs1799793 to clinical variables only for high threshold probability (Figure 2), even if it is necessary to underline that the obtained benefit seems not so crucial since the curves tend to overlap.

### Clinical-pathological features, germ-line polymorphisms and OS

The entire population of eligible patients (542 patients) was analyzed to evaluate the OS. The median follow-up of all patients, estimated from diagnosis until death by any cause or last follow-up, was 67 months (IQR: 45–94 months). One hundred seventeen of these patients (21.6%) died with a median follow-up of 71 months (IQR: 45–105 months). The overall 5- and 10-yr OS estimates were 89.9% and 59.7%, respectively.

**Figure 3:**
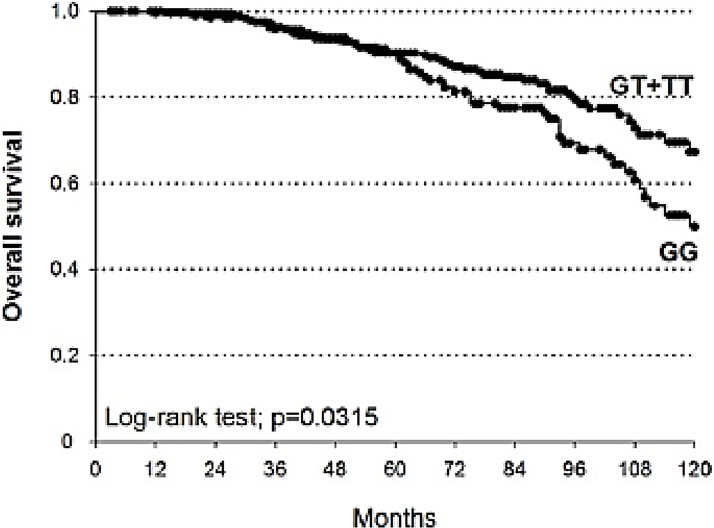
Kaplan-Meier estimates of OS at 10 years stratified according to genotypes of *MSH6*-rs3136228 (median survival: GT+TT = not reached; GG = 119 months)

The associations of clinical-pathological characteristics and polymorphisms with 5- and 10-yr OS were analyzed by multivariate Cox analysis (Table 4). After adjustment for Gleason score and PSA at diagnosis, only *MSH6*-rs3136228 was significantly associated with OS. In particular, the overall 5- and 10-yr survival for *MSH6*-rs3136228 GG patients were 89.5% and 49.9% respectively, while for patients carrying at least one T allele they were 90.3% and 67.3% (Log-rank test *p* = 0.0315; Figure 3). According to the dominant genetic model, patients carrying at least one T allele of *MSH6*-rs3136228 had an increased OS compared to patients bearing the GG genotype (HR = 0.63, 95%CI = 0.43–0.92, *p* = 0.0336).

**Table 4:** Univariate and multivariate Cox proportional hazards analysis of factors associated with overall survival

	OS (*n* = 542)					
	Univariate		Multivariate		Bootstrap analysis	
	HR (95% CI)	*p*-value	HR (95% CI)	*p*-value	HR (95% CI)	*p*-value
Gleason score						
2–6 (reference)						
7	0.88 (0.55–1.42)	0.6151	0.81 (0.49–1.33)	0.4050		
8–10	1.51 (0.96–2.32)	**0.0645**	1.20 (0.75–1.93)	0.4504		
PSA at diagnosis, ng/ml						
Continuous	1.004 (1.000–1.008)	**0.0568**	1.004 (0.999–1.008)	0.1000		
***MSH6* -rs3136228** Dominant model	0.68 (0.47–0.98)	**0.0383**	0.63 (0.43–0.92)	**0.0336**	**0.63 (0.41–0.98)**	**0.0405**

Abbreviations: OS = overall survival; PSA = prostate-specific antigen; HR = hazard ratio; CI = confidence interval; MSH6 = mutS homolog 6.

* bootstrap resampling method by drawing 1000 samples from the original dataset.

Significant associations (*p* < 0.05) are reported in bolt.

The *MSH6*-rs3136228 resulted significant also after bootstrap analysis (HR = 0.63, 95%CI = 0.41–0.98, *p* = 0.0405) (Table 3).

Nevertheless, in the sensitivity analysis it remained significant only in the subgroup of patients treated with high dosage of RT (> 70 Gy) and HT. Consequently, it seems that the dosage of RT could play a role in determining the prognostic role of this polymorphism (Table 2).

**Figure 4:**
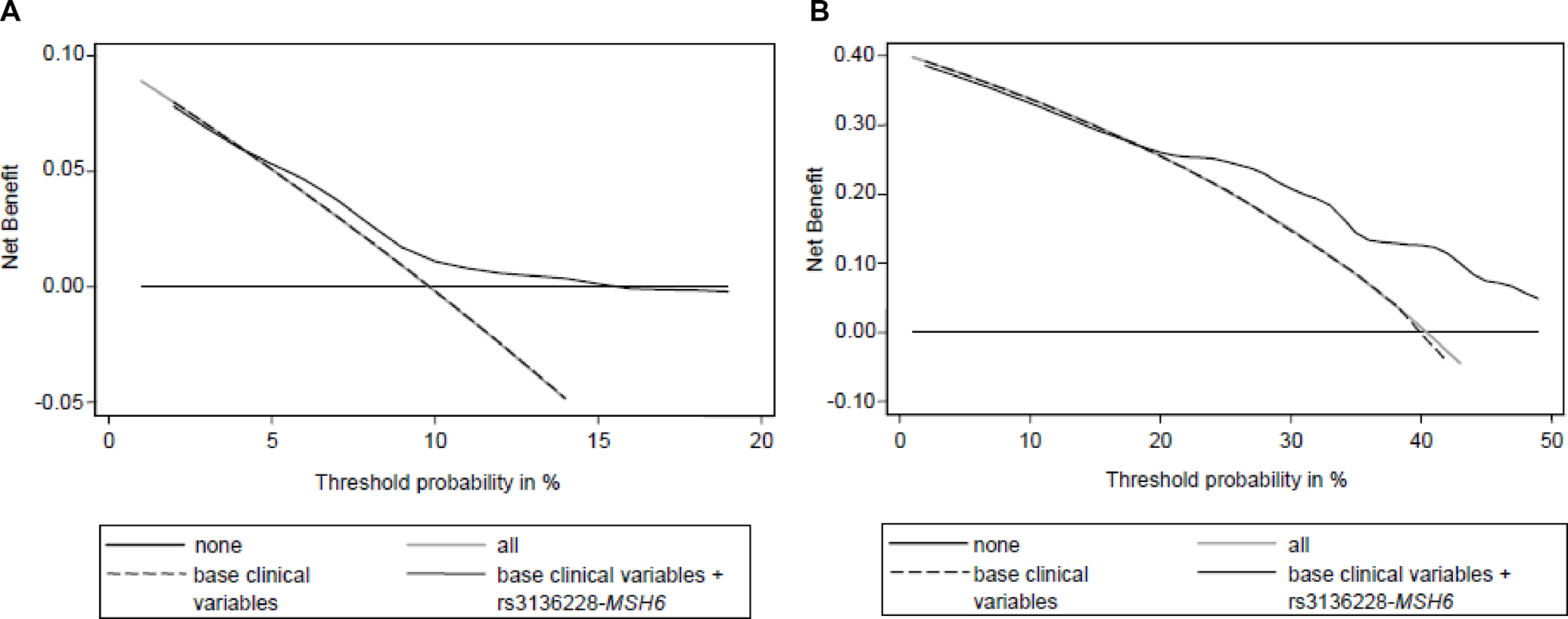
Decision curve analysis for OS at 5 years (**A**) and 10 years (**B**) including base clinical variables (Gleason score, serum PSA level at diagnosis, age at diagnosis, TNM stage, and RT dose (Gy)) and *MSH6*-rs3136228. “none”: no prognostic analyses are performed; “all”: hypothetical condition when you correctly define all patients that have the worst OS; “base”: only clinical variables are analyzed; “base+*MSH6*”: clinical variables and *MSH6*-rs3136228 are analyzed.

The DCA showed a considerable benefit adding the genetic analysis of *MSH6*-rs3136228 to the base clinical variables (Gleason score, serum PSA level at diagnosis, age at diagnosis, TNM stage, and RT dose (Gy)), for both 5-ys and 10-ys OS (Figure 4).

### Bioinformatic analysis

This analysis was performed to obtain some biological insights about the role of the three genetic variants highlighted by our analysis.

The first genetic variant of interest is *ERCC2*-rs1799793. The ERCC2 is an ATP-dependent DNA helicase belonging to the nucleotide excision repair (NER) pathway and in other cellular events like RNA transcription and chromosome segregation during mitosis. The *ERCC2*-rs1799793 is a missense variant that causes an amino acidic substitution in position 312 (Asp312Asn). Homozygous variant genotype (Asn/Asn-AA) show lower NER activity compared to homozygous wild-type genotype [16, 17]. CRAVAT analysis gave no ultimate response about the role of this polymorphism in this context. Specifically, it is not a cancer driver genetic variant. It has been associated with cisplatin treatment, not with RT. Additionally, mutations in ERCC2 have been observed not in PCa but in other settings (lung, ovary, stomach, large intestine, pancreas).

The second genetic biomarker is *EXO1-*rs4149963. According to UniProtKB database, EXO1 is a double-stranded DNA exonuclease involved in DNA mismatch repair (MMR), a member of the RAD2 nuclease family 5′->3′. It excises mismatch-containing DNA tracts directed by strand breaks located either 5′ or 3′ to the mismatch, through the direct interaction with other MMR proteins like MSH6 and MLH1. Moreover, it exhibits endonuclease activity and is involved in recombinational events too. No functional data are available up-to-date about *EXO1-*rs4149963. This is a missense polymorphism that causes the replacement of a polar amino acid (Thr) by a non-polar amino acid (Met) in position 439 of EXO1 protein. PolyPhen assigns a benignant role for this polymorphism. No functional predictions were available about polymorphisms in linkage with *EXO1-*rs4149963 according to SNPinfo web server. CRAVAT did not assign a cancer driving role to this polymorphism. Interestingly, mutations in EXO1 have been observed also in prostate cancer, highlighting the potential role of this gene for this malignancy.

Our study highlighted the potential clinical role also of *MSH6-*rs3136228. This protein is part of MMR. In particular, as reported by UniProtKB database, it forms a heterodimer with MSH2-MutS alpha. This complex then recognizes the DNA mismatches thereby initiating DNA repair and binding to other factors. The role of *MSH6-*rs3136228 was analyzed with transfection experiments: the variant G allele is associated with a reduction in promoter activity because it induces the loss of a the binding site of Sp1 transcription factor [18]. For this polymorphism, no results have been obtained with CRAVAT.

## DISCUSSION

The identification of prognostic biomarkers in PCa patients could exert a pivotal clinical role, optimizing patients’ treatment and management and substantially ameliorating the clinical course of this malignancy. Our study shed light to new potential genetic biomarkers: *ERCC2-*rs1799793 and *EXO1-*rs4149963, associated with BCR, and *MSH6*-rs3136228, associated with OS. Moreover, the clinical value of these new biomarkers have been explored with DCA and sensitivity analysis, even if no ultimate response has been obtained.

The primary aim of this study was the definition of new potential genetic biomarkers associated with PCa patients’ BCR

According to the dominant model, the presence of the variant allele of *ERCC2-*rs1799793 ensured a reduced risk to experience BCR, also after bootstrap analysis (HR = 0.58, 95%CI = 0.39–0.89, *p* = 0.0121). ERCC2 belongs to the NER pathway. Literature data have already addressed the potential association between genetic variants located in genes of NER pathway and response to RT in many kind of malignancies, such as head and neck cancer [19]. Moreover, the involvement of ERCC2 to other cellular events like RNA transcription and chromosome segregation could as well justify the obtained results.

As already stated, homozygous variant genotype (Asn/Asn-AA) show lower NER activity compared to homozygous wild-type genotype [16, 17]. At the best of our knowledge, no literature data concerning the role of *ERCC2-*rs1799793 on BCR in PCa in Caucasian population are available till now. Nonetheless, *ERCC2-*rs1799793 could be ascribed among PCa risk factors [20], as well as a predictive biomarker of response in various clinical settings [21–26], including RT [27, 28]. Considering the known biological function of this polymorphism, we can conclude that, even if no literature data are available about this polymorphism and the clinical problem we have investigated, the protective role of the A allele arose from our study is supported by some published data.

The second genetic biomarker associated with BCR was *EXO1-*rs4149963 (HR = 1.85, 95%CI = 1.16–2.98, *p* =0.0099). According to the dominant genetic model, the presence of the variant T allele of *EXO1-*rs4149963 was related with an increased BCR risk. This protein is involved in MMR, another pathway activated by RT. Specifically, MMR-related factors can bind to IR-induced DNA damages, promoting a G2/M cell cycle arrest and ultimately cell death by apoptosis [29].

At the best of our knowledge, only few literature data are available nowadays about *EXO1-*rs4149963. The presence of the variant allele seems to be associated with malignant conditions [30, 31]. There are no literature data about the functional role of this SNP that causes an aminoacidic substitution in position 439 (Thr439Met). Bioinformatic analysis gave us no ultimate results. Nonetheless, CRAVAT reported that mutations in EXO1 have been observed also in prostate cancer, highlighting the potential role of this gene for this malignancy. We can only hypothesize that this SNP, or others in linkage with it, could alter the interaction of EXO1 with other factors, potentially altering the efficiency of MMR and pathways involving EXO1.

A brief comment about the potentialities of introducing these genetic variants in the clinical practice is necessary. We have performed this evaluation with the sensitivity analysis and with the DCA. We applied the sensitivity analysis to assess the prognostic role of the significant biomarkers in subgroups of patients defined according the treatment peculiarities. Specifically, we grouped patients according to RT dosage and HT. Intriguingly, we obtained significant results for *ERCC2-*rs1799793 in all patients subgroups, underlining the strength of this biomarker and suggesting the potential clinical role of this polymorphism to predict BCR independently of the administered treatment. On the contrary, *EXO1-*rs4149963 was significant only in the subgroup characterized by high RT dosage (> 70 Gy). Thus, its prognostic role seemed to depend on the performed treatment, rendering it suitable only for specific patients subgroups. To going one step further, we applied the DCA to evaluate the reliability of predictions performed considering known prognostic clinical variables and of predictions that take into account both clinical variables and polymorhisms (*ERCC2-*rs1799793 and *EXO1-*rs4149963). The analysis of both *EXO1*-rs4149963 and *ERCC2*-rs1799793 seems to add a slight improvement to the analysis of clinical variables alone in predicting patients prognosis for high threshold probability. However, we have to acknowledge that, since the two curves tend to overlap, the benefit added by genetic information seems to be negligible.

Another aim of this study was the definition of new potential prognostic biomarkers associated with OS. According to the dominant model, patients bearing at least one variant T allele of *MSH6-*rs3136228 experienced a longer OS than patients carrying GG genotype (HR = 0.63, 95%CI = 0.41–0.98, *p* = 0.0405).

MSH6 takes part in MMR and it is directly involved in cancer onset due to its direct role in Lynch syndrome. As already mentioned, *MSH6-*rs3136228 is located in the gene promoter and influences the transcription efficiency [18]. It has been already associated with the risk to develop some cancers [32] and to an increased risk of neutropenia in colorectal cancer patients treated with fluoropyrimidines and oxaliplatin [33]. However, at the best of our knowledge, no significant evidences about the prognostic role of this polymorphism are currently available. The clinical significance of *MSH6-*rs3136228 has been investigated with the sensitivity analysis and with DCA. The sensitivity analysis reported that *MSH6-*rs3136228 remained significant only for the group treated with RT and HT. Comparing with the other two groups, it seems that RT dosage can have an impact on the obtained result. Nonetheless, according to the DCA, a considerable benefit was found adding *MSH6*-rs3136228 to the base model for both 5-and 10-ys OS.

Taken together, the data obtained from this study seem to stress the importance of MMR pathway in this clinical setting. It is indeed thought-provoking that proteins involved in MMR arose from both BCR and OS analyses. Thus, further studies focusing on this pathway could really pave the way to new interesting scenarios in this clinical setting.

We must acknowledge that this study presents some limitations. The most important limit is the lack of an independent validation set of patients. However, it is necessary to underline that the study population includes a large number of patients and that we have tried to overcome this limit introducing the bootstrap analysis as a kind of an internal validation. Another limit is that we are still not able to fully determine the potential clinical value of the identified biomarkers since the sensitivity analysis and the DCA did not let us to draw any final conclusion. Moreover, the definition of the threshold probability in DCA needs to be further discussed to correctly interpret it from a clinical point of view, as this is only an explorative analysis. Other considerations may come into play to determine whether or not genetic analysis could really play a clinical role in this scenario. An additional weak point of this study is represented by the difficulties in predicting the biological role of the genetic biomarkers. This for sure represents a topic that is necessary to better elucidate with at least *in vitro* studies.

To conclude, this single institutional study demonstrates that specific inherited variations in DRGs might be relevant predictors of BCR after RT (*ERCC2-*rs1799793 and *EXO1-*rs4149963) and OS (*MSH6-*rs3136228). Considering the obtained results, it is necessary to delve deeper into this clinical problem in order to optimize patients’ treatment and management, validating these results in a validation study.

## MATERIALS AND METHODS

### Patients’ recruitment and follow-up

Between 2003 and 2008 at CRO-National Cancer Institute, Aviano (Italy), 542 clinically localized PCa patients were enrolled. All underwent a primary RT based regimen, with or without HT. Eligibility criteria were the following: histologically confirmed diagnosis of primary PCa, Caucasian ethnicity, age ≥ 18 years, performance status (according to ECOG) 0–2, neutrophil count ≥ 1500/μL, platelet count ≥ 100000/μL, haemoglobin ≥ 9g/dL, total bilirubin < 1.5xULN (upper limit of normal), transaminase < 1.5xULN, creatinine < 1.5xULN, alkaline phosphatase < 2.0xULN. Patients were not affected by distal or nodal metastasis and should have a life expectancy higher than 6 months. Patients with no local disease control, impaired liver - (total bilirubin > 1.5xULN, ALT > 2xULN, AST > 2xULN) and renal function (creatinine > 1.5xULN), comorbidities that render not possible surgery or RT, relevant cardiovascular diseases (heart failure, acute myocardial infarction during the last year, angina in active phase, cardiac arrhythmia that need to be treated, not controlled hypertension) were excluded from the study.

Patients were treated with radical RT to the prostate, eventually in association with HT. Specifically, all patients underwent radical RT, at either at dosage ≤70Gy (47 patients −8.7%-) or > 70Gy (495 patients -91.3%-). Moreover, 466 patients (86.0%) underwent RT followed by HT and 76 patients (14.0%) were treated with RT alone.

Patients’ levels of serum PSA were monitored every three months for the first two years after the end of RT. The following monitoring was determined according to patients’ follow-up. According to the European Urology Association guidelines, BCR was defined for PSA levels higher than 2 ng/mL above the reached lowest level.

All patients signed a written informed consent for research purposes before entering this study, and all procedures were reviewed and approved by the institutional Review Board of CRO-National Cancer Institute.

### Patients’ treatment

All enrolled patients underwent RT. Due to the time span of enrolment, different treatment strategies were selected. Specifically, until 2006, external beam radiation therapy (EBRT) was three-dimensional (3D) with a 4- to 6-field technique and Linear Accelerator with 15-MV to 18-MV photon beams. The treatment planning was done in 3 phases with two different Planning Target Volumes (PTV). PTV1 includes prostatic bed and pelvic nodes. We used a four field box isocentric technique with anterior and posterior fields and left and right lateral fields. The AP-PA fields extended from interspace of first and second sacral vertebra to the ischial tuberosities and 1 cm outside the bony pelvis laterally. The parallel opposed 90° lateral fields encompassed the same dimensions longitudinally. The posterior border was set at the mid-lumen of the rectum and the anterior border was placed at the middle of the pelvic bone. The corners of the fields were trimmed to exclude the femoral heads, small bowel, posterior wall of the rectum and anus. The dose to the PTV1 was 26 Gy over 13 fractions (first phase) and after 2 weeks, with the same fields, 24 Gy over 12 fractions (third phase). PTV2 included only the prostatic bed treated with 6 fields and a dose of 20 Gy in 10 fractions (second phase). At the end, the prostatic bed was treated with 70 Gy over 35 fractions continuously while the pelvis was treated with 50 Gy over 25 fractions with split course of two weeks after 26 Gy. In setting of radical treatment, the strategy was the same in high risk patients with different doses. The dose and the fields of PTV1 was the same, while the dose of 6 fields to prostate (PTV2) was 30 Gy over 15 fractions. At the end the prostate was treated with 80 Gy over 40 fractions and nodes with 50 Gy over 25 fractions. In intermediate and low risk, the PTV included only prostate with a dose of 76 Gy over 38 fractions with 3D and 6 coplanar fields.

After 2006 all patients were treated with intensity-modulated RT (IMRT) with 15- to 18-MV photon beams. PTV)consisted of CTV plus a 5-mm margin in all directions except posteriorly, where a 3-mm margin was set. In patients with negative nodes, we usually used to treat external and internal iliac, obturator, with 50 Gy over 25 fractions, while with positive nodes the total dose, with same fields, was 56 Gy. A total dose of 70 Gy in 35 fractions was prescribed to prostatic bad. In radical settings, a local treatment directed to prostate was performed. Low and intermediate risk patients were treated with dose from 71,30 Gy over 31 fractions to 67,50 Gy over 27 fractions, while high risk patients underwent a RT dosage spanning from 70 Gy over 28 fractions to 75,90 Gy over 33 fractions. In 466 patients (86.0%) RT was administered in association with HT. According to international guidelines, high risk patients were treated with long term HT (2 or 3 years) based on an analogue of the luteinizing hormone-releasing hormone (LHRH). Intermediate risk patients underwent neoadjuvant 6 months HT with an analogue of LHRH or with bicalutamide 150 mg every day. In low risk patients affected by high prostate volume and thus candidate for HT, only bicalutamide 150 mg for 4 months in neoadjuvant setting was prescribed.

### Selection of polymorphisms and genotyping

Twenty-two polymorphisms in fourteen different DRGs were analyzed for this study. According to literature data, factors involved in different mechanisms of DNA repair were selected. Specifically, genes involved in NER (*ERCC1, ERCC2*), base excision repair (*APEX1, OGG1*), MMR (*EXO1, MLH1, MSH2, MSH6, MGMT*), single strand break repair (*PARP1, XRCC1*), and double strand break (*ATM*, *RAD51, XRCC3*) were included in this analysis. All these mechanisms were analyzed considering that RT can cause different kind of DNA damages.

We undertook a bibliographic search using PubMed and confining our analysis to studies performed in European-ancestry populations. We identified the publications using the combined keywords “radiotherapy”, “chemoradiotherapy’, “polymorphism”, “prostate cancer”, “cancer”, “pharmacogenetics” and the names of the previously identified genes. Genetic variants already analyzed in cancer patients undergoing RT have been selected, conferring a priority to those analyzed in PCa patients. Polymorphisms located in regions with a potential impact in protein activity or in gene expression have been included in the final list. We have thus selected polymorphisms located in the coding sequence (*APE1*-rs1130409, *ATM*-rs1800054, *APE1*-rs1801516, *ERCC1*-rs11615, *ERCC2*-rs13181, *ERCC2*-rs1799793, *EXO1*-rs4149963, *MLH1*-rs1799977, *OGG1*-rs1052133, *MGMT*-rs12917, *PARP1*-rs1136410, *XRCC1*-rs1799782, *XRCC1*-rs25487, *XRCC1*-rs25489, *XRCC3*-rs861539), in untranslated regions (UTRs) (*ERCC1*-rs3212986, *RAD51*-rs1801320, *XRCC1*-rs3213239, *XRCC3*-rs1799794), in regions potentially affecting mRNA splicing (*MSH2*-rs2303428, *XRCC3*-rs1799796) or potentially impacting gene regulation (*MSH6*-rs3136228). Supplementary Table 1 reports a list of pharmacogenetic studies, updated up to September 2016, analyzing the selected SNPs in cancer patients undergoing RT.

Biological samples of peripheral blood were collected from all enrolled patients. Genomic DNA was extracted with the QiaAmp DNA Mini Kit (Qiagen, Valencia, CA, USA) from peripheral blood and stored at +4°C until the time of this study.

For pyrosequencing analyses, the PCR primers were designed according to Primer3Plus (http://www.bioinformatics.nl/cgi-bin/primer3plus/primer3plus.cgi/). PCR amplifications were performed in an Eppendorf Mastercycler gradient, with TaqGold DNA Polymerase (AB Applied Biosystems, Warrington, UK). The analyses were exploited with PSQ96MA (Biotage, Uppsala, Sweden), using specific 5′-biotinylated primers. Predesigned TaqMan SNP genotyping assays were used for the allelic discrimination reactions. Analyses with TaqMan assays were performed with the Applera TaqMan Genotyping Master Mix on ABI 7900 HT instrument (AB Applied Biosystems, Foster City, CA, USA) according to the manufacturer instructions.

Positive and negative controls were always included in the analyses. For details about the position of polymorphisms in the gene, minor allele frequencies, genotype frequencies see Supplementary Table 2. Primers, assays, and PCR conditions are reported in Supplementary Table 3.

### Statistical analysis

The Hardy-Weinberg equilibrium was tested by a permutation procedure based on an exact test, claiming lack of agreement for *p* < 0.05.

The clinical end-points for this study were the BCR and OS, determined in both cases at 5- and 10-ys. The BCR was defined as the interval time between the end of RT and an increase of ≥ 2ng/mL above the serum lowest level. Regarding the analysis of OS, the interval time of interest spanned from the date of diagnosis to the date of death, or last known follow-up, whichever came first.

To analyze the association of polymorphisms with BCR or OS, Cox proportional hazards model was used. The analysis for BCR was adjusted according to age, Gleason score and PSA at diagnosis, meanwhile the covariates for OS were Gleason score and PSA at diagnosis. These clinical parameters were selected considering their known role in modulating BCR and OS. Dominant, recessive, and additive genetic models were considered for each polymorphism by combining heterozygous with homozygous genotypes. The best-fitting genetic model was selected according to Wald *X*^2^ test. Results were internally validated through a bootstrap resampling method by drawing 1000 samples from the original dataset. Bootstrap estimates of HRs, 95% confidence intervals (95% CI) and *p*-values were calculated. Survival analysis was computed by Kaplan-Meier method and log-rank test was used to test the differences between genotypes.

For both BCR and OS, a sensitivity analysis was performed in order to evaluate the strength of the obtained markers. More in detail, the polymorphisms resulted significant from multivariate analyses (*p* < 0.05) were also tested in three different subgroups of patients defined according to the treatment they underwent. In particular, one group included patients treated with RT and HT, another one only those treated with RT > 70Gy, and the last one only patients who underwent RT > 70Gy and HT. The numbers of the patients in the subgroups were different in BCR and OS due to the exclusion of 12 patients from OS analyses.

To evaluate the clinical advantage to introduce genetic information to the evaluation of clinical parameters to better stratify patients’ prognosis, DCA was performed according to the method described by Vickers [34]. In this graph, the curves were plotted in a Cartesian plane with “net benefit” on the vertical axis and “threshold probability” on the horizontal axis. The “threshold probability” can be defined as a level of certainty above which the patient would choose to perform the analyses that give the prognostic marker. This is related to efficacy, costs, adverse effects of such kind of analyses. The “net benefit” considered the difference between the expected benefit (i.e. number of true positives) and the expected harm (i.e. number of false positives multiplied by a weighting factor). A curve was drawn for each approach we explored. Specifically, one curve represented the application of the clinical variables and the other one the application of the clinical variables with the genetic data. A line termed as “none” was drawn to show what happens when no prognostic analyses are performed and thus no benefit can be achieved. Another line, “all”, represents the hypothetical condition when you correctly define all patients that experience relapse, in case of BCR, or death, in case of OS. For any probability threshold, the highest curve represents the optimal choice.

Cox regression analyses were performed with SAS 9.2 software, while DCA was determined with Stata 12 software.

### Bioinformatic analysis

To better understand the obtained results, we have deepened the role of the genetic variants not only with literature analysis but also with a bioinformatic approach. More in detail, the function of the genes was explored with UniProtKB database (http://www.uniprot.org/), whereas the potential role of the polymorphisms was studied with the exploitation of dbSNP (http://www.ncbi.nlm.nih.gov/SNP/), SNPinfo web server (http://www.ncbi.nlm.nih.gov/SNP/), PolyPhen (http://genetics.bwh.harvard.edu/pph2/). In addition, some tools of CRAVAT (https://www.cravat.us/CRAVAT/) were used to analyze both genes and polymorphisms. In particular, we defined the possible role of the genetic variants as cancer drivers and we analyzed the cancer sites characterized by mutations in the analyzed gene. All the obtained results are available upon request.

## SUPPLEMENTARY MATERIALS TABLES




