# Supplementary material for: Impact of DNA repair gene polymorphisms on the risk of biochemical recurrence after radiotherapy and overall survival in prostate cancer
Source: Oncotarget. 2017 Feb 11;8(14):22863–75. doi: 10.18632/oncotarget.15282 (PMC5410269; doi:10.18632/oncotarget.15282)
Supplement: Supplementary file 1 [file oncotarget-08-22863-s001.pdf]

# Impact of DNA repair gene polymorphisms on the risk of biochemical recurrence after radiotherapy and overall survival in prostate cancer

## Supplementary Materials

**Supplementary Table 1: Studies assessing the influence of polymorphisms in DNA repair genes on clinical outcome after radiotherapy−based treatment**

| Gene | SNP | Cancer | Number of patients | End−point | References |
|---|---|---|---|---|---|
| *APE1* | rs1130409 | Rectum | 280 | Treatment response | [35] |
| *ATM* | rs1800054 | Prostate and breast | 637 prostate and 967 breast | Radiation induced adverse response | [36] |
| | rs1801516 | Prostate | 698 | Radiation induced gastrointestinal and genitourinary acute toxicity | [37] |
| *ERCC1* | rs3212986 | Rectum | 238 | Treatment response | [38] |
| | rs11615 | Prostate | 494 | Cancer progression | [39] |
| *ERCC2* | rs13181 | Prostate | 494 | Cancer progression | [39] |
| | rs1799793 | Prostate | 698 | Radiation induced gastrointestinal and genitourinary acute toxicity | [37] |
| *EXO1* | rs4149963 | Rectum | 280 | Treatment response | [35] |
| *MLH1* | rs1799977 | Prostate | 698 | Radiation induced gastrointestinal and genitourinary acute toxicity | [37] |
| *MSH2* | rs2303428 | Rectum | 238 | Treatment response | [38] |
| *MSH6* | rs3136228 | Rectum | 280 | Treatment response | [35] |
| *OGG1* | rs1052133 | Rectum | 238 | Treatment response | [38] |
| *MGMT* | rs12917 | Breast | 87 | Skin reaction after RT | [40] |
| *PARP1* | rs1136410 | Prostate | 284 | Overall survival | [7] |
| *RAD51* | rs1801320 | NSCLC | 228 | Overall survival and radiation pneumonitis | [41] |
| *XRCC1* | rs1799782 | Prostate | 494 | Cancer progression | [39] |
| | rs3213239 | Rectum | 280 | Treatment response | [35] |
| | rs25487 | Prostate | 494 | Cancer progression | [39] |
| | rs25489 | Prostate | 494 | Cancer progression | [39] |
| *XRCC3* | rs861539 | NSCLC | 228 | Overall survival and radiation pneumonitis | [41] |
| | rs1799794 | Prostate | 698 | Radiation induced gastrointestinal and genitourinary acute toxicity | [37] |
| | rs1799796 | Rectum | 280 | Treatment response | [35] |

APE1=Apurinic/apyrimidinic endonuclease 1; ATM40=Ataxia Telangiectasia Mutated 40; ATM61=Ataxia Telangiectasia Mutated 61; ERCC1=Excision Repair Cross−Complementing 1; ERCC2=Excision Repair Cross−Complementing 2; EXO1= Exonuclease 1; MLH1= mutL homolog 1; MSH2= mutL homolog 2; MSH6= mutS homolog 6; OGG1= 8−Oxoguanine glycosylase; MGMT=O(6)−methylguanine−DNA methyltransferase; PARP1=poly (ADP−ribose) polymerase 1; RAD51=RAD51 recombinase; XRCC1= X−ray Repair Cross−complementing Group 1; XRCC3= X−ray Repair Cross−complementing Group 3; NSCLC= non small cell lung cancer

**Supplementary Table 2: List of selected polymorphisms, minor allele frequencies (MAF), and genotype frequencies**

| Gene | SNP | Amino acid change | Location | Major/minor allele | MAF | Genotype frequencies |
|---|---|---|---|---|---|---|
| *APE1* | rs1130409 | Asp148Glu | missense | G/T | 45.16 | GG: 168 (31.28%) GT: 253 (47.11%) TT: 116 (21.60%) |
| *ATM* | rs1800054 | Ser49Cys | missense | C/G | 1.21 | CC: 525 (97.58%) CG: 13 (2.42%) GG: 0 (0.00%) |
| | rs1801516 | Asp1853Asn | missense | G/A | 13.86 | GG: 406 (75.05%) GA: 120 (22.18%) AA: 15 (2.77%) |
| *ERCC1* | rs3212986 | 3′UTR | 3′UTR | T/G | 26.07 | TT: 293 (54.36%) TG: 211 (39.15%) GG: 35 (6.49%) |
| | rs11615 | Asn118Asn | cds−synon | T/C | 40.02 | TT: 197 (36.41%) TC: 255 (47.13%) CC: 89 (16.45%) |
| *ERCC2* | rs13181 | Lys751Gln | missense | G/T | 40.54 | GG: 185 (34.32%) GT: 271 (50.28%) TT: 83 (15.40%) |
| | rs1799793 | Asp312Asn | missense | G/A | 34.32 | GG: 244 (45.27%) GA: 220 (40.82%) AA: 75 (13.91%) |
| *EXO1* | rs4149963 | Thr439Met | missense | C/T | 9.12 | CC: 444 (82.68%) CT: 88 (16.39%) TT: 5 (0.93%) |
| *MLH1* | rs1799977 | Ile219Val | missense | A/G | 34.38 | AA: 231 (42.70%) AG: 248 (45.84%) GG: 62 (11.46%) |
| *MSH2* | rs2303428 | − | intronic | T/C | 9.61 | TT: 441 (81.52%) TC: 96 (17.74%) CC: 4 (0.74%) |
| *MSH6* | rs3136228 | − | 5′UTR | G/T | 38.64 | GG: 195 (36.31%) GT: 269 (50.09%) TT: 73 (13.59%) |
| *OGG1* | rs1052133 | Ser326Cys | missense | C/G | 19.81 | CC: 344 (63.70%) CG: 178 (32.96%) GG: 18 (3.33%) |
| *MGMT* | rs12917 | Leu115Phe | missense | C/T | 17.07 | CC: 377 (69.94%) CT: 140 (25.97%) TT: 3 (0.55%) |
| *PARP1* | rs1136410 | Val762Ala | missense | T/C | 15.52 | TT: 384 (71.38%) TC: 141 (26.21%) CC: 13 (2.42%) |
| *RAD51* | rs1801320 | 5′UTR | 5′UTR | G/C | 8.76 | GG:451 (83.21%) GC: 87 (16.05%) CC: 4 (0.74%) |
| *XRCC1* | rs1799782 | Arg194Trp | missense | C/T | 9.35 | CC: 444 (82.22%) CT: 91 (16.85%) TT: 5 (0.93%) |
| | rs3213239 | − | intronic | −/GGCC | 39.46 | Del/Del: 201 (37.50%) Del/Ins: I247 (46.08%)ns/Ins: 88 (16.42%) |
| | rs25487 | Gln399Arg | missense | G/A | 34.38 | GG: 236 (43.62%) GA: 238 (43.99%) AA: 67 (12.38%) |
| | rs25489 | Arg280His | missense | G/A | 7.05 | GG: 470 (87.20%) GA: 62 (11.50%) AA: 7 (1.30%) |
| *XRCC3* | rs861539 | Thr241Met | missense | C/T | 41.22 | CC: 187 (34.57%) CT: 262 (48.43%) TT: 92 (17.01%) |
| | rs1799794 | 5′UTR | 5′UTR | A/G | 18.30 | AA: 355 (65.62%) AG: 174 (32.16%) GG: 12 (2.22%) |
| | rs1799796 | − | intronic | A/G | 30.15 | AA: 258 (47.87%) AG: 237 (43.97%) GG: 44 (8.16%) |

APE1 = Apurinic/apyrimidinic endonuclease 1; ATM40 = Ataxia Telangiectasia Mutated 40; ATM61 = Ataxia Telangiectasia Mutated 61; ERCC1 = Excision Repair Cross-Complementing 1; ERCC2 = Excision Repair Cross−Complementing 2; EXO1 = exonuclease 1; MLH1= mutL homolog 1; MSH2 = mutL homolog 2; MSH6= mutS homolog 6; OGG1 = 8−Oxoguanine glycosylase; MGMT = O(6)-methylguanine−DNA methyltransferase; PARP1 = poly (ADP-ribose) polymerase 1; RAD51 = RAD51 recombinase; XRCC1= X-ray Repair Cross-complementing Group 1; XRCC3= X-ray Repair Cross−complementing Group 3; cds= coding sequence; SNP =single nucleotide polymorphism; UTR = untranslated region; synon = synonymous; Del = deletion; Ins = insertion

**Supplementary Table 3: Details of genotyping methods.** See Supplementary_Table_3
